# Conservative Management of a Slow-Flow Orbital Cavernous Hemangioma: A Case Report

**DOI:** 10.7759/cureus.72309

**Published:** 2024-10-24

**Authors:** Zainab Habbash

**Affiliations:** 1 Radiology, Salmaniya Medical Complex, Manama, BHR

**Keywords:** conservative management, mri, orbital cavernous hemangioma, orbital tumor, proptosis, slow-flow vascular lesion

## Abstract

Orbital cavernous hemangiomas are the most common benign orbital tumors in adults, typically presenting as slowly progressive, painless proptosis. These lesions can lead to cosmetic concerns, optic nerve compression, and, in severe cases, visual impairment. Timely diagnosis and appropriate management are crucial. This case report presents the diagnostic approach, management, and follow-up of a patient with a slow-flow orbital cavernous hemangioma, adhering to conservative management principles.

A 37-year-old woman presented with a six-month history of progressive left eye proptosis without visual disturbances or pain. Physical examination revealed non-pulsatile proptosis with normal visual acuity and no optic nerve involvement. Laboratory investigations, including thyroid function tests, were unremarkable. Orbital ultrasonography revealed a well-defined hypoechoic mass and subsequent magnetic resonance imaging confirmed a 2.5 cm, lobulated, hyperintense lesion in the left orbit, consistent with an orbital cavernous hemangioma. The lesion was extraconal, displacing but not compressing the optic nerve. A diagnosis of a slow-flow orbital cavernous hemangioma was made. Orbital cavernous hemangiomas can be effectively managed conservatively in cases with minimal symptoms and no visual impairment. Regular follow-up is essential to monitor for progression, and surgical intervention should be reserved for cases with significant proptosis or optic nerve compression.

## Introduction

Orbital cavernous hemangiomas are the most common benign vascular tumors of the orbit in adults, typically presenting as slowly progressive, painless proptosis. These lesions are composed of dilated vascular channels lined by endothelial cells, and they are usually located within the intraconal or extraconal spaces of the orbit [1-4]. Although cavernous hemangiomas are congenital, they often remain asymptomatic until later in life when they begin to enlarge. The exact cause of their growth remains unclear, but factors such as hormonal changes or local hemodynamic forces may play a role.

Clinically, patients often present with unilateral, non-pulsatile proptosis, which is gradually progressive. Visual acuity is usually preserved unless the tumor causes compression of the optic nerve or other orbital structures [1,3]. Imaging, particularly magnetic resonance imaging, plays a crucial role in the diagnosis, as these lesions have characteristic features such as well-defined, lobulated margins and hyperintensity on T2-weighted images [2,5].

Management of orbital cavernous hemangiomas depends on the size of the lesion, associated symptoms, and the degree of visual impairment. While asymptomatic cases may be managed conservatively with regular follow-up, surgical excision is often considered for cases with significant proptosis, optic nerve compression, or cosmetic concerns. 

## Case presentation

A 37-year-old, right-handed female presented to the ophthalmology clinic with a six-month history of gradually worsening left eye proptosis. She first noticed a slight bulging of her left eye, which she initially dismissed, but over time, the eye protrusion became more prominent and associated with mild discomfort. There was no history of trauma, visual disturbances, or systemic symptoms such as fever, weight loss, or night sweats. Her past medical history was unremarkable, with no history of chronic illness or prior orbital disease. She denied any history of thyroid dysfunction, autoimmune disease, or recent infections. The patient was otherwise healthy, with no significant family history of ocular or systemic diseases.

On physical examination, the patient was alert and cooperative, with vital signs within normal limits. Ocular inspection revealed noticeable proptosis of the left eye, approximately 4 mm compared to the right, measured using Hertel’s exophthalmometer. The proptosis was non-pulsatile and non-tender to palpation. No periorbital swelling or erythema was noted. The patient had normal extraocular muscle movements, and there was no diplopia. Visual acuity was 20/20 bilaterally, with normal color vision and pupillary reflexes. Anterior segment examination of both eyes was unremarkable. Fundoscopy showed no evidence of optic disc swelling or retinal abnormalities. The remainder of the physical examination was unremarkable, with no signs of systemic involvement or lymphadenopathy.

Given the progressive nature of the proptosis and the absence of systemic signs or trauma, a comprehensive workup was initiated. Laboratory investigations, including complete blood count (CBC), thyroid function tests, and inflammatory markers (erythrocyte sedimentation rate (ESR) and C-reactive protein (CRP)), were all within normal ranges. Thyroid-stimulating hormone (TSH) and free T4 levels were also normal, effectively ruling out thyroid eye disease as a potential cause.

**Figure 1:**
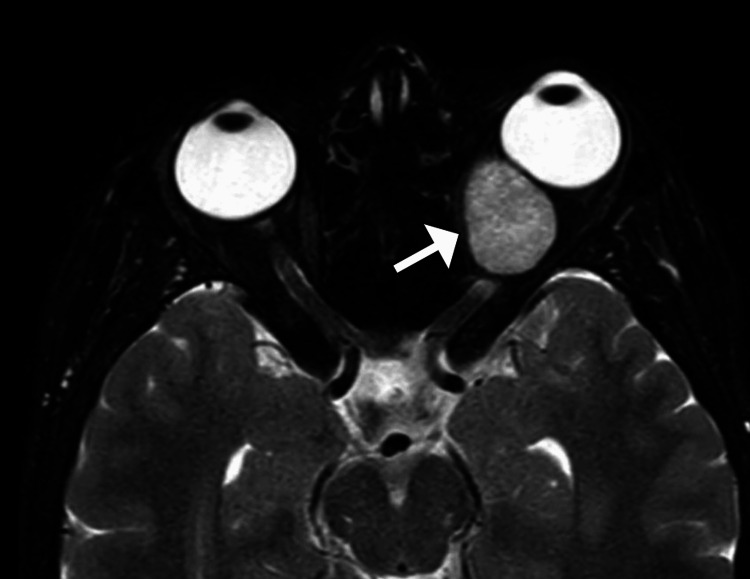
Axial MRI T2-weighted image demonstrating a well-defined, hyperintense intraconal lesion in the left orbit, consistent with a cavernous hemangioma (arrow) The lesion is causing mild proptosis without evidence of optic nerve compression or infiltration of adjacent structures.

**Figure 2:**
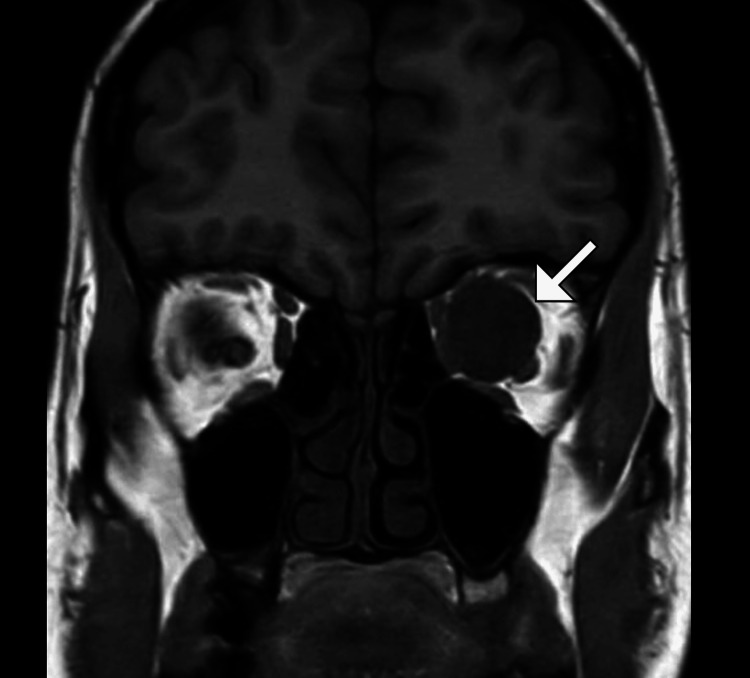
Coronal MRI T1-weighted pre-contrast image showing a well-circumscribed, hypointense, intraconal lesion (arrow) in the left orbit The lesion is located medial to the optic nerve, without evidence of invasion or compression of surrounding structures, consistent with a cavernous hemangioma.

**Figure 3:**
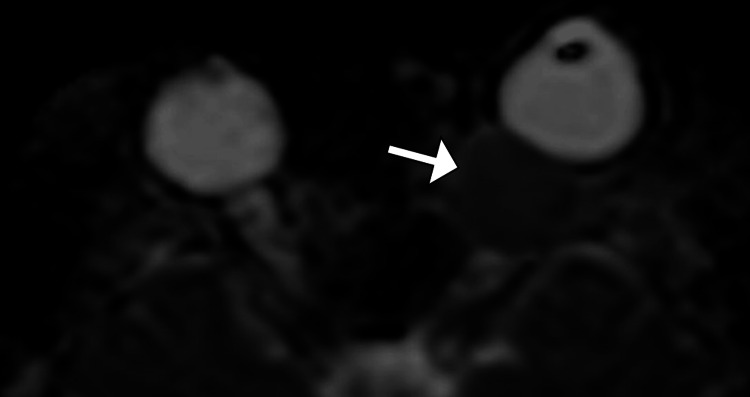
Axial apparent diffusion coefficient (ADC) map of the orbits demonstrating the left intraconal lesion (arrow) with low signal intensity, indicative of restricted diffusion, consistent with the characteristics of a slow-flow cavernous hemangioma

**Figure 4:**
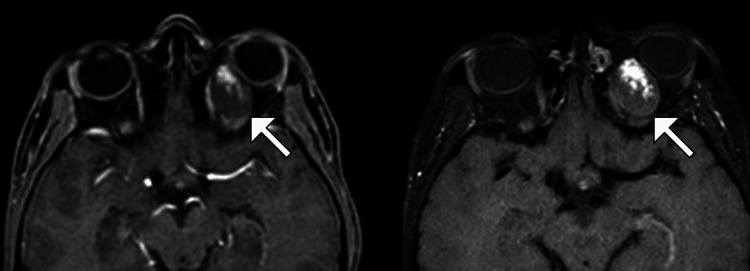
Axial T1-weighted MRI with contrast showing a well-defined left intraconal lesion with heterogeneous progressive enhancement (arrows) The lesion is displacing adjacent orbital structures, including the optic nerve, but without evidence of direct invasion, consistent with the imaging characteristics of a cavernous hemangioma.

Imaging studies were essential in further delineating the cause of the proptosis. Initial orbital ultrasonography revealed a well-defined, hypoechoic lesion located within the left orbit, suggestive of a vascular mass. Subsequently, magnetic resonance imaging (MRI) of the orbits with contrast was performed, which demonstrated a well-circumscribed, lobulated mass measuring approximately 2.5 cm in the superomedial quadrant of the left orbit. The lesion was hyperintense on T2-weighted images and showed heterogeneous enhancement after gadolinium administration. The mass was located extraconally, displacing the optic nerve inferiorly but without direct involvement or compression. These imaging characteristics were highly suggestive of a cavernous hemangioma, a benign vascular lesion commonly seen in the orbit (Figures 1-4).

The differential diagnosis at this stage included cavernous hemangioma, orbital lymphangioma, and less likely, a slow-flow arteriovenous malformation (AVM). However, given the imaging findings, the absence of systemic symptoms, and the slow progression of the lesion, a slow-flow orbital cavernous hemangioma was considered the most likely diagnosis.

After a multidisciplinary discussion, the patient was diagnosed with a slow-flow cavernous hemangioma of the left orbit. The decision was made to manage the patient conservatively, as her symptoms were mild and her visual function remained intact. Regular follow-up with serial imaging was recommended to monitor for any increase in size or worsening of symptoms that would necessitate intervention. The patient was counseled about the benign nature of the lesion and the potential for surgical excision should the mass enlarge or cause significant functional impairment.

During her hospital course, the patient remained stable, and no acute interventions were required. She was discharged with instructions to follow up in three months. At her most recent follow-up, 12 months post-diagnosis, the patient remained asymptomatic, with no increase in proptosis or development of visual disturbances. The slow-flow cavernous hemangioma remained stable, and no surgical intervention was deemed necessary. She continues to be monitored regularly for any potential progression.

## Discussion

Orbital cavernous hemangiomas are the most common benign orbital tumors in adults, comprising approximately 4-7% of all orbital masses. Although these lesions are typically slow-growing and asymptomatic, their clinical relevance lies in the potential for progressive proptosis, optic nerve compression, and cosmetic deformity, making timely diagnosis and appropriate management essential [2-5]. This case of a slow-flow orbital cavernous hemangioma highlights several important aspects of diagnosis, management, and considerations for therapeutic intervention.

The patient’s gradual onset of painless proptosis without visual impairment is a typical presentation of orbital cavernous hemangiomas. These tumors are non-infiltrative, encapsulated, and located in either the intraconal or extraconal space, with most lesions identified incidentally or after causing mass effect within the orbit. The absence of systemic or inflammatory symptoms in this case, as well as the normal thyroid function tests, effectively ruled out differential diagnoses such as thyroid eye disease, lymphoma, and orbital pseudotumor. Orbital imaging, especially with MRI, was crucial in confirming the diagnosis. The characteristic imaging features of cavernous hemangiomas - well-circumscribed, hyperintense on T2-weighted MRI, and showing enhancement after gadolinium contrast - were pivotal in distinguishing this lesion from other orbital masses such as lymphangiomas or arteriovenous malformations (AVMs) [1,6]. While AVMs may present similarly, the slow-flow nature of the cavernous hemangioma in this case was well-demonstrated by the absence of significant vascular flow on imaging.

A key point in the management of orbital cavernous hemangiomas is the decision between conservative observation and surgical excision. Historically, surgical removal has been the treatment of choice for symptomatic cases, particularly those causing optic nerve compression, diplopia, or significant cosmetic disfigurement. The benign nature of the lesion, however, means that aggressive treatment is not always warranted [1-3]. In our case, the patient’s mild symptoms and the stability of her visual function and imaging findings justified a conservative approach. This approach is supported by recent literature, which emphasizes the importance of individualized management based on symptomatology and the risk-benefit ratio of surgical intervention. Studies have shown that asymptomatic or mildly symptomatic cavernous hemangiomas can often be managed conservatively with periodic clinical follow-up, as was the case here. This strategy reduces the risks associated with orbital surgery, such as diplopia, enophthalmos, or optic nerve damage, which may outweigh the benefits in cases without functional impairment.

Surgical management, while effective in resolving proptosis and alleviating compressive symptoms, is not without risks. Orbital cavernous hemangiomas are often located in proximity to critical structures, including the optic nerve, extraocular muscles, and globe. As such, even with advancements in microsurgical techniques and access to specialized orbital surgeons, there remains a risk of intraoperative complications. A tailored, patient-centered approach, as demonstrated in this case, aligns with the current trend toward conservative management in asymptomatic or minimally symptomatic cases. The multidisciplinary input in this case, including ophthalmology, radiology, and neurosurgery, highlights the importance of a collaborative approach in managing orbital masses, particularly those with complex anatomical considerations [2-4].

Follow-up remains a cornerstone of the management of orbital cavernous hemangiomas, particularly given the potential for slow but progressive growth. Although this case showed stability over 12 months of follow-up, longer-term surveillance is warranted, as late growth or symptom development may necessitate reconsideration of the therapeutic approach. Recent studies suggest that the growth rate of these tumors is often minimal, and in many cases, they remain stable over years. However, there are reports of rapid enlargement, particularly during periods of hormonal fluctuation, which emphasizes the need for ongoing monitoring [4-6].

This case also highlights the potential future avenues for research into orbital cavernous hemangiomas. The pathophysiology underlying their growth remains poorly understood, and further studies into the molecular and vascular mechanisms driving their expansion may open new therapeutic avenues. While the role of medical therapies, such as corticosteroids or anti-angiogenic agents, remains largely unexplored in this context, there is growing interest in their potential for managing vascular malformations in other parts of the body. Whether these therapies may play a role in the treatment of orbital cavernous hemangiomas remains an open question that warrants further investigation.

## Conclusions

In conclusion, this case illustrates the classical presentation and imaging features of a slow-flow orbital cavernous hemangioma and underscores the importance of individualized management strategies. A conservative approach, as employed here, can be highly effective for asymptomatic or mildly symptomatic cases, avoiding the risks of surgery while ensuring careful long-term follow-up. Further research into molecular biology and alternative treatment strategies for orbital hemangiomas could provide valuable insights into more targeted and less invasive management options in the future. The management of orbital cavernous hemangiomas remains a dynamic field, with ongoing advancements in both diagnostic imaging and surgical techniques, but the focus on patient-centered, evidence-based care remains paramount.
